# Predicting Species Distributions Using Record Centre Data: Multi-Scale Modelling of Habitat Suitability for Bat Roosts

**DOI:** 10.1371/journal.pone.0128440

**Published:** 2015-06-08

**Authors:** Chloe Bellamy, John Altringham

**Affiliations:** School of Biology, Faculty of Biological Sciences, University of Leeds, Leeds, United Kingdom; University of Porto, PORTUGAL

## Abstract

Conservation increasingly operates at the landscape scale. For this to be effective, we need landscape scale information on species distributions and the environmental factors that underpin them. Species records are becoming increasingly available via data centres and online portals, but they are often patchy and biased. We demonstrate how such data can yield useful habitat suitability models, using bat roost records as an example. We analysed the effects of environmental variables at eight spatial scales (500 m – 6 km) on roost selection by eight bat species (*Pipistrellus pipistrellus*, *P*. *pygmaeus*, *Nyctalus noctula*, *Myotis mystacinus*, *M*. *brandtii*, *M*. *nattereri*, *M*. *daubentonii*, and *Plecotus auritus*) using the presence-only modelling software MaxEnt. Modelling was carried out on a selection of 418 data centre roost records from the Lake District National Park, UK. Target group pseudoabsences were selected to reduce the impact of sampling bias. Multi-scale models, combining variables measured at their best performing spatial scales, were used to predict roosting habitat suitability, yielding models with useful predictive abilities. Small areas of deciduous woodland consistently increased roosting habitat suitability, but other habitat associations varied between species and scales. *Pipistrellus* were positively related to built environments at small scales, and depended on large-scale woodland availability. The other, more specialist, species were highly sensitive to human-altered landscapes, avoiding even small rural towns. The strength of many relationships at large scales suggests that bats are sensitive to habitat modifications far from the roost itself. The fine resolution, large extent maps will aid targeted decision-making by conservationists and planners. We have made available an ArcGIS toolbox that automates the production of multi-scale variables, to facilitate the application of our methods to other taxa and locations. Habitat suitability modelling has the potential to become a standard tool for supporting landscape-scale decision-making as relevant data and open source, user-friendly, and peer-reviewed software become widely available.

## Introduction

Urban expansion, transport development and the intensification of agricultural, industrial and forestry practices continue to change the landscape. The result is reduced and fragmented natural habitats with declining biodiversity and impaired ecosystem services (e.g. [1][2]) that probably lack resilience to the pressures of climate change, pests and diseases, and human disturbance. With ‘natural’ areas reduced to small and isolated fragments in an increasingly hostile matrix, protection must involve not only maintaining these fragments, but also increasing the ecological connectivity between them and improving the landscape mosaic in which they sit [3]. This need for a ‘landscape scale’ conservation strategy is now universally acknowledged [4]. Effective management on this scale requires knowledge of the distribution of the species of concern and of the ways that this is determined by environmental factors, both those we can control and those we cannot. Tools are therefore required to assess the distribution of habitats and species, and to forecast the impacts of environmental change. Habitat Suitability Modelling (HSM, or Species Distribution Modelling, SDM) enables species distributions to be predicted over large areas from environmental data and species occurrence records. It has been usefully applied using few presence-only data (e.g. [5]), giving it considerable potential in practical conservation, since large presence/absence datasets are frequently unavailable or unreliable and species records are becoming increasingly accessible from record centres and online data portals.

The obvious benefit of using existing databases to address ecological questions is the time and money saved in gathering new data. However, such data present challenges to creating useful HSMs because they are often of a coarse spatial resolution, lack important metadata, may be out of date, and can suffer from error and geographic and environmental sampling bias [6]. Models calibrated with biased presence data and random pseudoabsences may reflect the skewed sampling effort within the species data, rather than the environment with which the species’ presence is truly correlated [7][8]. Methods to check and select species datasets, and model with small [5] or biased [7][9] presence-only datasets, have been developed. For example, records of other species that have been collected using similar sampling methods to the species being modelled (and are therefore likely to be equally biased towards certain environments) can be used as pseudoabsences, instead of a random selection of locations across the study area [7]. This “target group approach” has been shown to reduce the impact of sampling bias on predictions and improve model performance [7][9][10], although it assumes an equal likelihood of the detection of all species in all environments sampled [8].

We investigated whether the HSM approach could be used to accurately predict roosting habitat suitability for eight species of bat (*P*. *pipistrellus*, *P*. *pygmaeus*, *P*. *auritus*, *M*. *daubentonii*, *M*. *nattereri*, *M*. *brandtii*, *M*. *mystacinus*, and *N*. *noctula*) at a fine resolution across the Lake District National Park, NW England, using record centre data. Bat roosts are legally protected in Britain and the sensitivity of these mammals to habitat change makes them good indicators of biodiversity and general environmental conditions [11]. In temperate regions bats can spend up to twenty hours of their day in roosts during the breeding period, typically in trees and built structures. Roosts therefore play a vital role in bats’ lives, and the selection of a roost site will affect survival and reproductive success [12].

The suitability of a potential roost will be determined not only by the characteristics of the roost itself, but by the composition and structure of the surrounding landscape (e.g. [13][14][15]. Long flights are energetically expensive and bats should therefore select roosts with suitable foraging and drinking sites, alternative day roosts, night roosts, hibernacula, and interconnecting commuting routes, within distances appropriate to the species’ ecology and behaviour [12]. We used the presence-only modelling software MaxEnt [16][17] to study how characteristics of the surrounding landscape affect roost site selection and to predict habitat suitability for roosting bats across the entire Park. The results offer insights into each species’ habitat associations, distribution, niche breadth and conservation status, as well as providing spatially explicit, landscape scale decision-making tools for conservationists and planners.

## Materials and Methods

### Roost records

**Fig 1:**
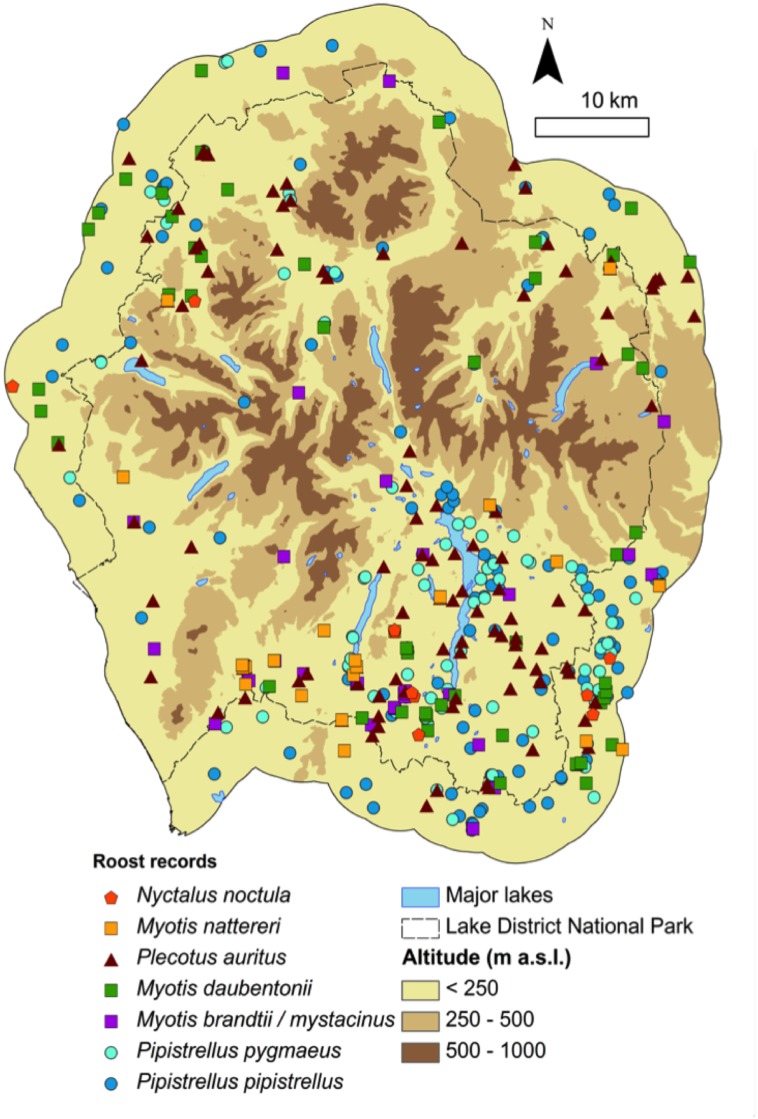
Map of the species’ roost records used from the Lake District National Park, NW England. Sample size: *P*. *pipistrellus—129; P*. *pygmaeus—80; N*. *noctula—10; M*. *brandtii / mystacinus—24; M*. *daubentonii—51; M*. *nattereri—23; P*. *auritus—102*. NB. Drawn at this scale, overlap between roosts masks many species’ roost locations. See Fig 5 for separate species’ roost maps. Crown database right 2010. An Ordnance Survey/EDINA supplied service.

The study area chosen was the Lake District National Park (~2,300 km^2^) in NW England. It has wide post-glacial valleys filled with a rich mosaic of woodland, plantations, lakes, farmland and open grassland, turning into moorland and rocky, mountainous terrain at higher elevations (up to 977 m a.s.l.). Built areas vary from scattered farms linked by unlit tracks to small towns and well-lit, busy roads. Georeferenced bat roost records from across Cumbria were supplied by the Cumbria Biodiversity Data Centre (www.cbdc.org.uk). 3,891 records made between 1980 and 2009 were provided, including roost observations of eight species: *Pipistrellus pipistrellus*, *P*. *pygmaeus*, *Plecotus auritus*, *Myotis daubentonii*, *M*. *nattereri*, *M*. *brandtii*, *M*. *mystacinus*, and *Nyctalus noctula*. The records were provided by naturalists, local bat groups and other organisations, and a small number of records were added from incidental fieldwork by the authors (S2 File). The detail supplied on the type of bat activity recorded, its location, and the methods used to identify the species, varied between records. The database was filtered to select species-specific, summer day roost records. All data were georeferenced (≤100m resolution) using the location details provided by recorders. *P*. *pipistrellus* records pre-1998, which may have included misidentifications of *P*. *pygmaeus* [18], were excluded. Records were retained only if the species was identified “in the hand” or from appropriate bat detector recordings, at a day roost, April to September inclusive. *M*. *brandtii* and *M*. *mystacinus*, which are cryptic in terms of both their morphology and echolocation calls, were grouped together because species records are likely to have included a high proportion of misidentifications. Duplicate records were removed. Roosts were not differentiated according to their size or use (e.g. maternity roosts) since this information was not available for every record. Finally, roosts in the Lake District National Park and a surrounding 5 km buffer (subsequently referred to as the “study area”; Fig 1) were extracted for analysis. Development in the National Park is strictly regulated to protect the region’s character and environment, so there has been minimal change to landcover and land use since its designation in 1951. Records from all years provided (1980–2009 or 1998–2009 for *Pipistrellus*) were therefore used, since it was considered unlikely that the surroundings of the roost site had changed significantly since the time the record was made. The available records almost certainly underestimate the use of trees as roosts by some species, so our study has a probable bias towards roosts in built structures, a feature common to many similar studies.

### Pseudoabsences

In an attempt to reduce the impact of sampling bias on the model predictions, pseudoabsences were carefully selected, rather than allowing MaxEnt to select them randomly from across the study area, as is the default. This was done based on the records available and our previous knowledge of the structures the species predominantly roost in:

1. Mammal record pseudoabsences. For those species that have been observed roosting in a wide range of structures, including trees, woodland bat boxes, bridges, tunnels and buildings (S2 File), a target group approach was taken [7]. All post-1980 mammal species records (including bats) from the database recorded with a resolution of ≤100 m were used as the target group because these records were assumed to have been collected using a similar sampling strategy by naturalists. To reduce the impact of species richness influencing an area’s estimated sampling effort [19] data were filtered so that only one record remained per 100 m square, which left 9,826 mammal records (S1 File). This number is close to the 10,000 recommended by Phillips & Dudík [17] to maximise MaxEnt performance.
2. Building location pseudoabsences. For those species that roost predominantly in buildings (S2 File), we identified the centre-point of all buildings across the study area larger than 10 x 10 m using the OS MasterMap Topography data. These locations were filtered so that we had just one record per 100 x 100 m square. From this dataset, 10,000 building locations were selected at random (S1 File). Only building roost records were used from the species data. The models therefore inform us of differences between the habitat surrounding roost buildings and a random selection of buildings across the study area.

### Environmental data

GIS data from multiple sources were used to create gridded environmental variables (100 m resolution rasters; Table 1; S1 File). GIS manipulation was performed in ArcGIS 10.0 (www.esri.com). In brief, two non-scalar environmental variables were used in each model (distance to water and woodland edge) and all other variables were measured at eight scales (500, 1,000, 1,500, 2,000, 3,000, 4,000, 5,000 and 6,000 m) by measuring cell statistics within different sized windows centred on each raster cell using the focal statistics tool. This multi-scale approach provides important information on the direction and strength of relationships between a species and characteristics of the surrounding environment over a range of scales that is typical of a species’ home range, and enables the production of more powerful models compared to a single-scale approach [14][15]. Full details on the GIS methods are given in [15] and S1 File, along with an ArcGIS toolbox “MultiScaleMaxent” that automates this process.

**Table 1:** The fifteen habitat variables used for analysis. All layers were produced at 8 different spatial scales except the two distance variables.

GIS data layer	Description	Original data source
Distance to inland water (m)	Euclidean distance to nearest inland water feature	OS MasterMap Topography Layer
Distance to woodland edge (m)	Euclidean distance to nearest woodland edge	OS MasterMap Topography Layer
Majority aspect (categorical: flat, N, NE, E, SE, S, SW, W, NW)	Majority aspect at multiple scales	OS Land-Form PROFILE DTM
Mean altitude (m a.s.l.)	Mean altitude at multiple scales	OS Land-Form PROFILE DTM
Mean slope (°)	Mean slope at multiple scales	OS Land-Form PROFILE DTM
Cover of inland water (%)	Percentage cover of inland water at multiple scales	OS MasterMap Topography Layer
Cover of deciduous wood (%)	Percentage cover of deciduous wood at multiple scales	OS MasterMap Topography Layer
Cover of coniferous wood (%)	Percentage cover of coniferous wood at multiple scales	OS MasterMap Topography Layer
Cover of mixed wood (%)	Percentage cover of mixed wood at multiple scales	National Inventory of Woodland & Trees & OS MasterMap Topography Layer
Cover of buildings (%)	Percentage cover of buildings at multiple scales	OS MasterMap Topography Layer
Cover of manmade surfaces (%)	Percentage cover of manmade surfaces and structures at multiple scales	OS MasterMap Topography Layer
Cover of ancient wood (%)	Percentage cover of (non-replanted) ancient wood at multiple scales	Ancient Woodland Inventory (Provisional) for England
Habitat richness (%)	Proportion of 13 habitat types present at multiple scales	OS MasterMap Topography Layer
Maximum woodland patch (km^2^)	Size of the largest woodland patch within or intersecting the different sized scale windows	OS MasterMap Topography Layer
Woodland edge density (km/km^2^)	Length of woodland edge per unit area at multiple scales	OS MasterMap Topography Layer

### Modelling

#### Model fitting

MaxEnt is a program that predicts the geographical distribution of a species based on the environmental conditions at locations where the species is known to occur, using the maximum entropy method [16]. All models were run in MaxEnt Version 3.3.3e (http://www.cs.princeton.edu/~schapire/maxent), using primarily default settings and one species record per cell (see S1 File). The predictive power of a variable was measured at each individual scale by building and testing univariate models using 5-fold cross validation (≥25 roost records), or leave-one-out jackknife validation (<25 records). In addition, we measured the average and maximum distances between the species’ roosts and woodland edge or inland water and compared these to the pseudoabsences data.

#### From univariate to multivariate models

All environmental variables measured were first tested for their performance in a univariate species model at each spatial scale. The scale at which an environmental variable had the highest test AUC in a univariate model was entered into a species’ multivariate model, to create ‘full models’ of all variables at their best performing scale. Environmental variables with test AUC ≤0.5 or test gain ≤0.01 were removed and those remaining were checked for multicollinearity (r ≥0.70) using ENMTools (www.ENMTools.com; [20][21]). Collinear variables were pruned by retaining those with higher test AUC scores. Models were further reduced to yield a set of variables with the highest predictive power, using a jackknife, leave-one-out stepwise approach [15][22]. Environmental variables causing the smallest decrease (or largest increase) in test AUC when removed were pruned in turn until only one environmental variable under consideration remained. The ‘pruned model’ with the highest test AUC was used for further analysis. For species with small sample sizes (<25) we chose the variable set with the lowest extrinsic omission rate and associated *P*-value, but if multiple variable sets generated equally low values, we chose the model with the highest test AUC. The contribution each remaining environmental variable made to a species’ pruned model was determined by randomly permuting the value of each environmental variable in turn at the presence and pseudoabsence locations and re-testing the model. The resulting, normalised drop in training AUC was compared between variables [23].

The fraction of the entire study area that was predicted to be suitable roosting habitat for each species was calculated using the occupancy threshold rule that maximises the sum of test sensitivity and specificity, which is recommended for presence-only modelling [24]. Extrinsic omission rates (the proportion of test points that fall outside this suitable area) and their statistical significance were calculated (using a binomial test) to aid assessment of model performance. Residual spatial autocorrelation (rSAC) can inflate measures of model performance [15][25][26][27] therefore Moran‘s correlograms were created (1 – predicted HSI for each species record; 25) using Spatial Analysis in Macroecology (SAM, http://www.ecoevol.ufg.br/sam; [28]). Significance of Moran’s I was calculated using a randomisation test with 9,999 Monte Carlo permutations, correcting for multiple testing. If significant residual spatial autocorrelation (rSAC) was detected we spatially aggregated the species data so that the distances between the five data subsets were maximised [15][22], and performance measures were re-calculated using 5-fold cross validation.

The entire set of a species’ roost records were then combined to generate final models and roosting habitat suitability maps for the study area. The habitat suitability maps produced display predictions of how suitable an area is for a roosting bat, regardless of whether suitable roost structures were available.

Niche breadth and species richness were also studied and the methods and results are described in S1 File and S2 File.

## Results

Of the 3,891 bat records for Cumbria (1980–2009), 730 records were selected for modelling (S2 File), of which 418 fell within the study area. The most frequently recorded species were *Pipistrellus pipistrellus*, *P*. *pygmaeus* and *Plecotus auritus*. After categorising roosts (building, tree, bat box, bridge or other structures) we were able to identify which set of pseudoabsences would be most appropriate for each species (full details in S2 File). The building pseudoabsences chosen for *P*. *pipistrellus*, *P*. *pygmaeus* and *P*. *auritus*, *Myotis brandtii* and *M*. *mystacinus*, since ≥90% of roosts were in buildings. Only ten roost records were available for *Nyctalus noctula*, located in tree holes, bat boxes and a telegraph pole. *Myotis daubentonii* was recorded roosting primarily in bridges and *M*. *nattereri* in range of manmade structures (buildings, bridges and bat boxes). We therefore selected the mammal target group pseudoabsences for *M*. *nattereri*, *M*. *daubentonii* and *N*. *noctula*. We trained and tested the *M*. *nattereri*, *M*. *brandtii/mystacinus* and *N*. *noctula* models using the jackknife approach because of the low number of records available (<25).

### Univariate species-habitat associations

#### Distance to water and woodland edge

**Fig 2:**
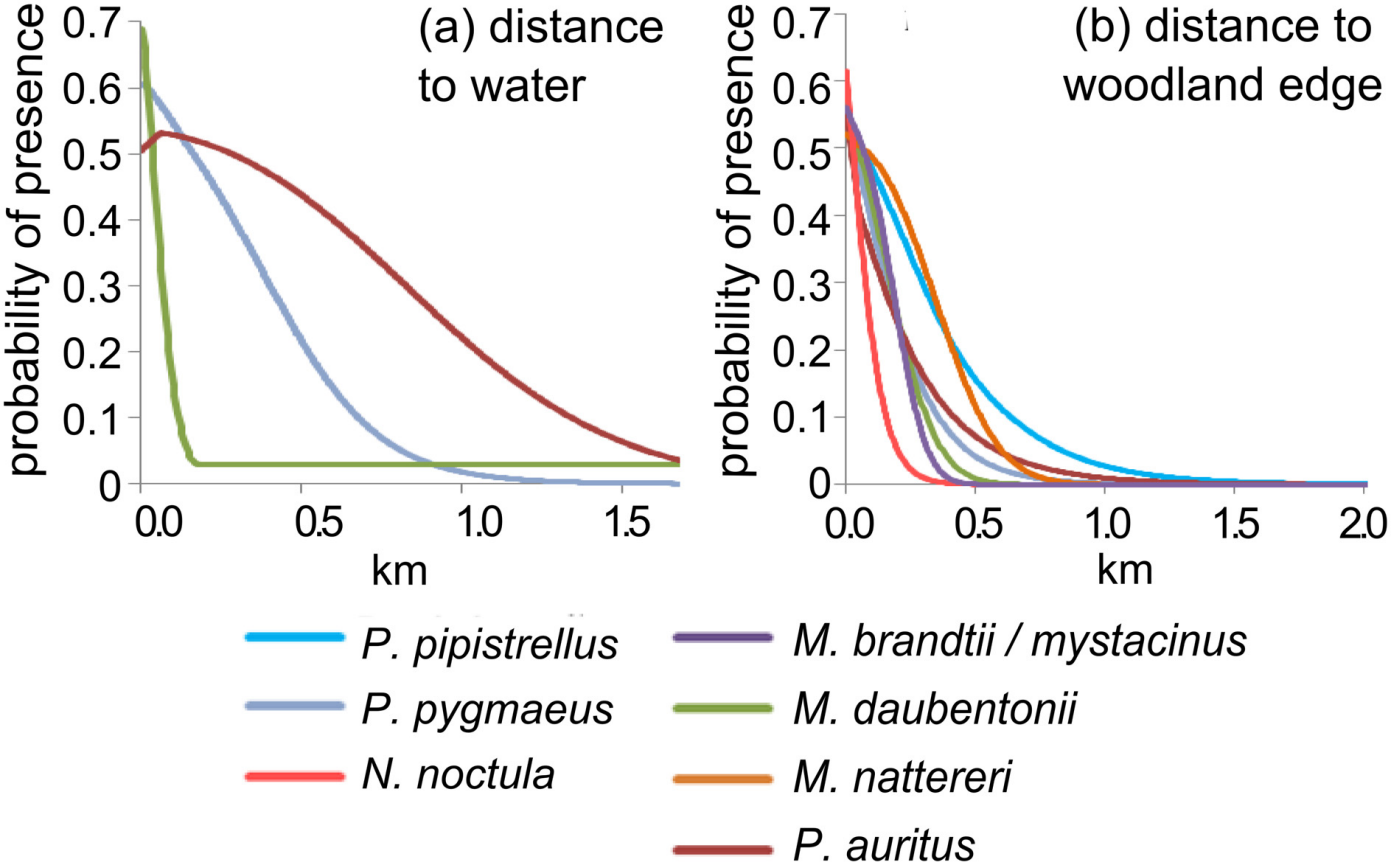
Distance to water and woodland edge response curves. These graphs show probability of a species’ roost *(p)* at a location based on these distances and are based on the results of univariate models to prevent any other interacting or collinear variables affecting the relationships found. Variables which were found to have poor predictive power for a species (AUC ≤ 0.5 or test gain < 0.01) are not shown.

Probability of roost presence increased with proximity to water and woodland, but the strength and slope of relationships varied between species (Fig 2). The mean distance between a bat roost and woodland edge was < 50 m for all species, apart from *P*. *pipistrellus* (65 m). This compares to a mean distance of 104 m between woodland edge and random buildings (S2 File). This strong association is evident in the MaxEnt response curves (Fig 2): most response curves declined to a probability of zero at distances greater than 1,000 m. There was more interspecific variation in distances between roosts and inland water (S2 File) and this variable was useful only for predicting the presence of *M*. *daubentonii*, *P*. *pygmaeus*, and *P*. *auritus* (Fig 2). *M*. *daubentonii* roosts were closest on average to water (42 m) and the probability of finding a roost declined sharply between 0 and 200 m. This decline was less steep for *P*. *pygmaeus* and less steep still for *P*. *auritus* (Fig 2).

#### Species-habitat relationships at different scales

**Fig 3:**
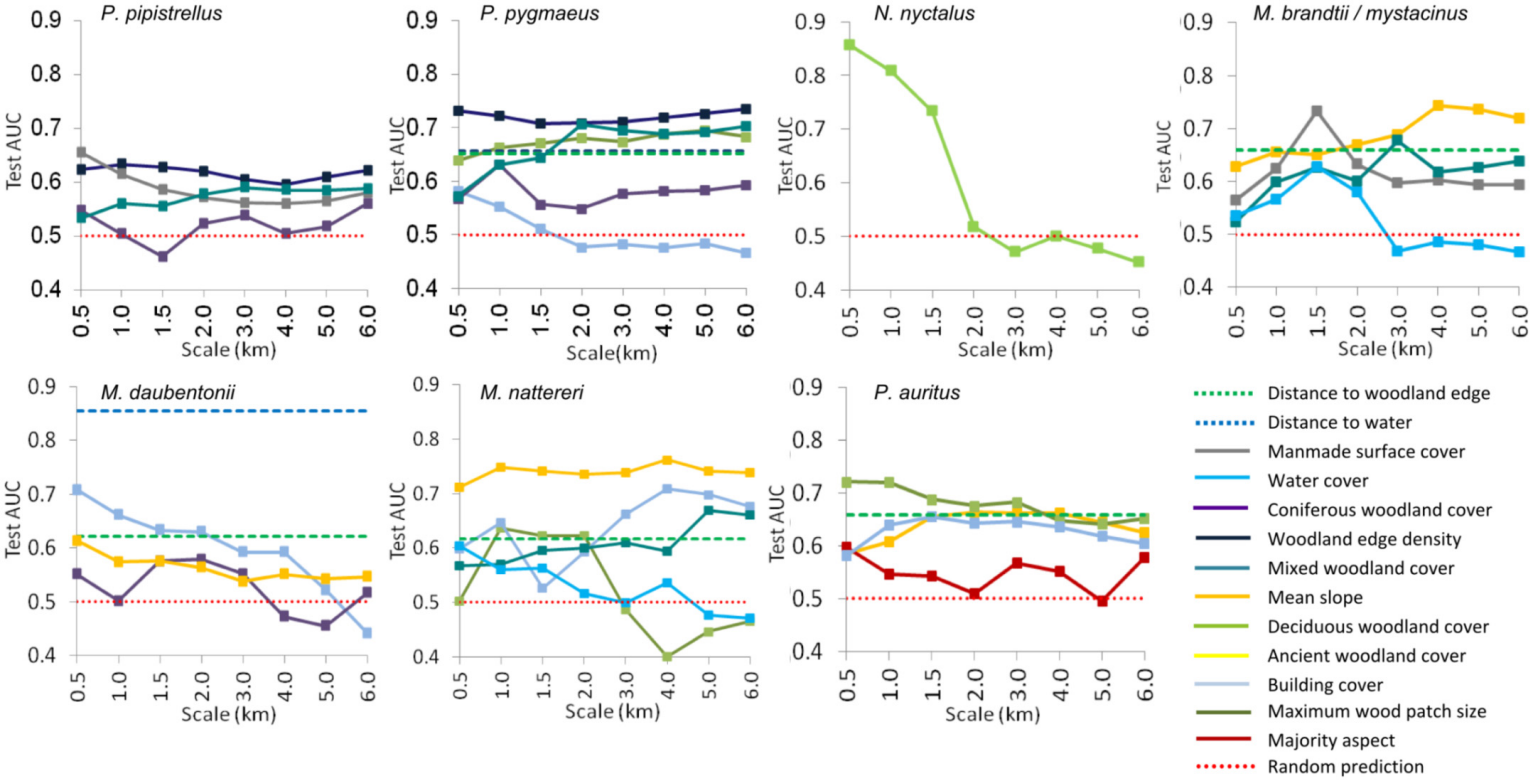
Variable performance. These graphs show the strength of association (as test AUC) between each species’ presence and individual environmental variables at different spatial scales. The average predictive power of the distance variables is shown as a dashed line: these were independent of scale. Environmental variables with a predictive power ≤ 0.5 are no better than random. Only variables retained in pruned models are shown. NB. The scale range is not linear for improved clarity at small scale.

Fig 3 shows the strength of association (test AUC as an estimate of predictive power) from univariate models, across the scales, for each species. Only the variables retained in the final, multivariate pruned models are shown. For most species many variables maintained similar test AUC scores between 500 and 6,000 m scales. The exception was *N*. *noctula*, whose presence was predominantly associated with variables measured at large spatial scales (>2,000 m). The habitat associations found are too numerous to describe for all scales and species, therefore only selected results are reported.

### Multivariate models

#### Model performance

Threshold independent measures indicated that models performed well on average (test AUC 0.71–0.89; test gain ≥0.29; Table 2). Extrinsic omission rates were also low (range: 0.00–0.23, Table 2). These were all significantly lower than expected by chance alone for species with sample sizes large enough for 5-fold cross validation. Significant rSAC was detected in the *P*. *pipistrellus*, *P*. *pygmaeus*, *M*. *nattereri* and *P*. *auritus* pruned models. Model residuals were positively autocorrelated up to a distance lag of 7–16 km, depending on the species. Those models with sample sizes >25 were re-run using spatially constrained 5-fold cross validation. Test AUC and gain dropped (by 0.07±0.02 and 0.36±0.15 respectively), and training AUC and extrinsic omission rates increased slightly (by 0.004±0.001 and 0.04±0.09 respectively), as expected (mean ± S.D.; Table 3). However, omission rates remained significant and test AUC values were 0.65–0.72. Therefore, the best final models, having spatially constrained test data where necessary and possible, had test AUCs of 0.65–0.89.

**Table 2:** Mean model performance. Measured using random 5-fold cross validation *(P*. *pipistrellus*, *P*. *pygmaeus*, *M*. *daubentonii and P*. *auritus)*, or jackknife validation *(N*. *noctula*, *M*. *brandtii/mystacinus and M*. *nattereri)* tests.

Species	Train set	Test set	Train AUC	Test AUC	Test gain	Test omission rates
*P*. *pipistrellus*	103.2	25.8	0.740±0.02	0.713±0.05	0.285±0.17	0.233***
*P*. *pygmaeus*	63.2	15.8	0.835±0.01	0.802±0.05	0.620±0.20	0.137***
*N*. *noctula*	9	1	0.889±0.02	0.865±0.12	0.848±0.78	0.000
*M*. *brandtii / mystacinus*	23	1	0.897±0.01	0.829±0.05	0.687±0.64	0.000
*M*. *daubentonii*	40.8	10.2	0.910±0.01	0.888±0.05	1.143±0.37	0.040***
*M*. *nattereri*	22	1	0.858±0.01	0.801±0.25	0.562±1.23	0.000
*P*. *auritus*	81.6	20.4	0.804±0.02	0.769±0.05	0.344±0.45	0.198***

Train set = average number of training data; Test set = average number of test data; Test omission rates = average proportion of test data which fell outside of the suitable area. Omission rates which are significantly lower than expected by chance alone are in bold type. Asterisks signify level of significance (*** p<0.001), all values ± S.D.

**Table 3:** Mean model performance from spatially constrained 5-fold cross validation. rSAC lag = largest distance within which data pairs retained significant, positive spatial autocorrelation.

Spp.	rSAC lag (km)	Mean (km)	Min (km)	rSAC pairs (%)	Train set	Test set	Train AUC	Test AUC	Test gain	Test omission
*P*. *pipistrellus*	8	29.1 ±16.7	0.08	7.6	103.2	25.8	0.746 ±0.03	0.650 ±0.12	0.013 ±0.41	0.168*
*P*. *pygmaeus*	7	24.0 ±15.3	0.10	8.1	64.8	16.2	0.839 ±0.02	0.714 ±0.11	0.088 ±0.60	0.197*
*P*. *auritus*	16	27.5 ±14.9	0.12	29.7	81.6	20.4	0.807 ±0.03	0.718 ±0.14	0.082 ±0.76	0.264*

Mean = the mean distance between all spatially aggregated training and test data pairs; Min = the minimum distance between spatially constrained training and test data pairs; rSAC pairs = the percentage of all spatially constrained training and test data pairs that still fell within the rSAC lag; Train set = average number of training data; Test set = average number of test data; omission rates = average proportion of test data which fell outside of the suitable area; omission rates which are significantly lower than expected by chance alone are in bold type. Asterisks signify level of significance (* p<0.05); all mean values ± S.D.

#### Model composition

**Fig 4:**
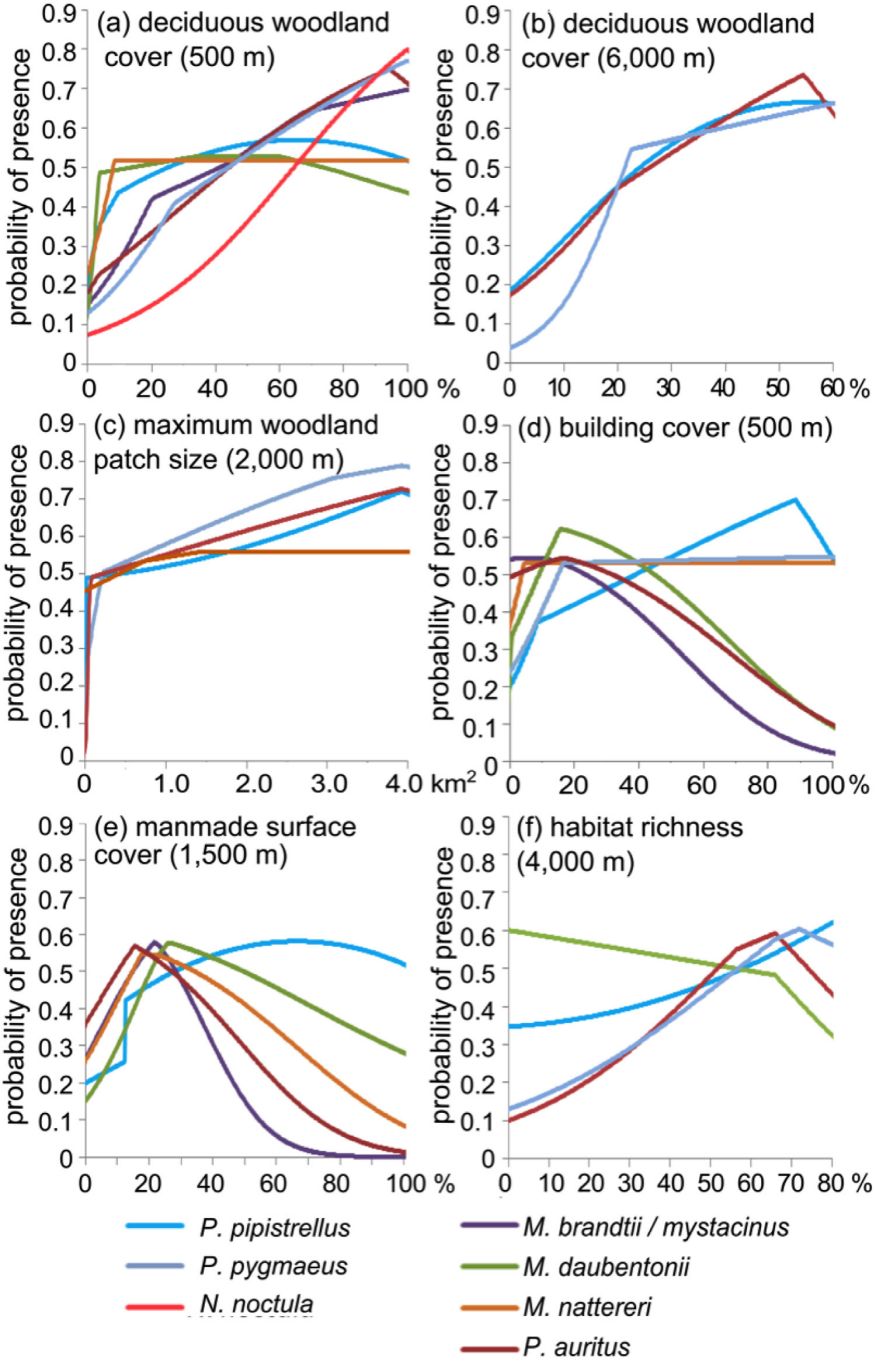
Representative MaxEnt response curves. These graphs show the probability of a species’ presence at a location for a range of parameters. These graphs are based on univariate models to prevent interacting or collinear variables from affecting the relationships modelled. Variables found to have poor predictive power for a species (AUC ≤ 0.5 or test gain < 0.01) are not shown.

**Fig 5:**
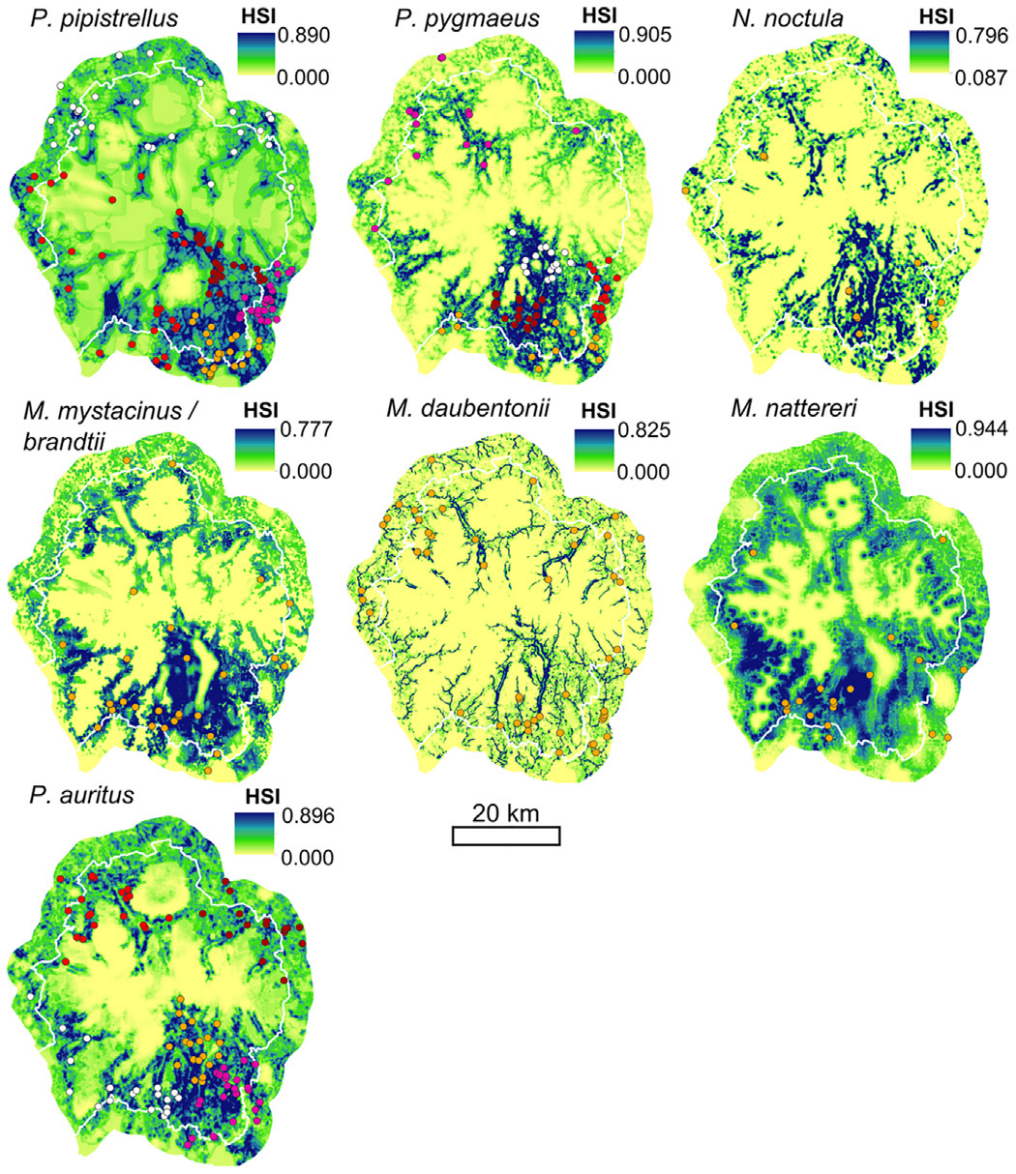
Habitat suitability maps made using each species’ pruned set of variables. HSI = Habitat Suitability Index. The Lake District National Park boundary is marked in white. Species roost locations are coloured by test subsets where appropriate. Crown database right 2010. An Ordnance Survey/EDINA supplied service.

After pruning, multivariate models contained one to seven variables, with unique sets of variables being retained in each species’ model (Figs 3 and 4), resulting in species-specific patterns of suitability across the Park (Fig 5). Full details of the models, including marginal response curves and the contribution each variable makes to the model for each species, are summarised in S2 File. The spatially explicit results were overlaid and compared in ArcGIS and ENMTools, illustrating the degree to which species’ niches overlapped, highlighting “hotspots” of shared habitat and providing information on niche breadth (S2 File).

Only deciduous woodland cover at the 500 m scale remained in the *N*. *noctula* model, which had a positive effect on roosting habitat suitability. The *M*. *daubentonii* model also only contained variables measured at the 500 m scale, in addition to distance to water and woodland edge. Proximity to water contributed most to this model (69%), limiting this species’ highly suitable habitat to areas within 150 m of water. Good *M*. *daubentonii* roosting habitat therefore followed the paths of rivers, streams and lake edges. Within 500 m of these suitable areas, a lack of woodland edge, large areas of coniferous woodland or building cover, or the presence of steep slopes caused a decline in the roosting habitat suitability index.

*P*. *pipistrellus* roost presence was best predicted by manmade surface cover and woodland edge density at small scales. Mixed woodland cover at the 3,000 m scale further increased habitat suitability, but areas with more than 30% coniferous woodland cover at the 6,000 m scale were avoided. Highly suitable habitats were concentrated around settlements and roads close to woodland edge, especially areas with large areas of mixed woodland. Coniferous woodland cover also reduced *P*. *pygmaeus* roosting habitat suitability, but at the 1,000 m scale. *P*. *pygmaeus* selected areas close to water and woodland edge, with high densities of woodland edge and large patches of woodland available within 5,000–6,000 m of the roost (Fig 4).

The presence of *M*. *brandtii/mystacinus* roosts was positively correlated with proximity to water and woodland edge. However, they avoided roosting in areas with high water cover within 2,000 m, and dense manmade surface cover within 1,500 m. In addition, they selected for areas of medium slope (4,000 m), with some mixed woodland (3,000 m). *M*. *nattereri* had similar habitat associations to *M*. *brandtii/mystacinus*. It also favoured woodland edge in areas with medium slopes (4,000 m) but avoided built up areas and high water cover. *P*. *auritus* also avoided built up areas (1,500 m) and favoured high deciduous woodland cover and locations with large woodland patches within 500 m (Fig 4). It also favoured gentle slopes averaged over 2,000 m and westerly aspects at the 500 m scale. All maps reflected an association with wooded areas. A shared avoidance of built up areas created pockets of habitat unsuitability around the small towns in the Park. Poor roosting habitat also existed around the large lakes for *M*. *nattereri* and *M*. *brandtii/mystacinus*.

## Discussion

### Model performance

We found that with appropriate measures to minimise bias MaxEnt can generate roosting habitat suitability models with useful levels of accuracy from existing record centre data. The models predict suitability for roosting over large areas, regardless of the availability of specific roost sites. Both AUC and extrinsic omission rates were used to assess performance [29], given the concerns in applying AUC alone to validate presence-only models [30]. Residual spatial autocorrelation (rSAC) in some models caused a slight inflation of performance measures, which may be explained by the colonial nature of bats and the shared use of multiple roost sites. Model performance may have been improved by tuning MaxEnt settings such as the regularisation multiplier [31], however, previous research has indicated that the regularisation multiplier of two used in this study was optimal for other fine resolution bat-habitat suitability models [15][32]. Future work should focus on increasing our understanding how parameter tuning and pseudoabsence selection affect variable selection and model performance [29][31][33]. To address the potential problems in using record centre data to create habitat suitability models [6], we adopted conservative methods. We carefully selected species records, used a target group approach in an attempt to counteract potential sampling bias, and measured the impact of rSAC on model performance. We selected only summer roost data because of the different habitat requirements of bats during migration, swarming, and hibernation. The period (April – September) will incorporate some variability in behaviour and subsequent roost selection. Ideally, separate models should be developed for particular behaviours or groups of individuals that have distinctive habit requirements (e.g. age, lifecycle or sex categories) to provide more targeted information to practitioners [34]. However, this is frequently neither possible nor practical. The selection process used to identify the most reliable and relevant records will depend on the quality and quantity of data provided and in most cases it will be necessary to relax selection rules to ensure the reduction in sample size does not greatly affect model power.

Interpretation and application of those models built for species with small sample sizes (<25) should be carried out with caution. Despite applying a jackknife approach to building and assessing models (as recommended by [5][29], these models tend to be less reliable as they are highly sensitive to each individual record used [5]. The *N*. *noctula* model (n = 10), which included only one environmental variable following variable pruning, is less useful and informative compared to the other species’ models. It is therefore advisable to collect field data in cases where low record centre sample sizes preclude the production of a useful model. However, this is often not possible, particularly for rare or inconspicuous species. Finally, there are other potential pitfalls and reasons to be cautious in making inferences about species habitat relations from MaxEnt [8][35], but with careful use, our results show that it can be a valuable tool.

### Species-habitat relationships and the importance of scale

In a parallel study, we found that the suitability of habitat for foraging bats was best predicted by small scale and distance variables, reflecting their ability to exploit small habitat patches [15]. Roost sites, however, must provide foraging and drinking areas, night roosts and alternative day roosts within a colony’s home range, and connect these sites. The varied distribution and availability of these resources across a landscape and over time should therefore result in the selection of a roost site according to habitat composition and structure over a greater range of scales than selection of foraging sites. This is precisely what we found. For example, *Pipistrellus* foraging habitat suitability could only be predicted by woodland edge density when measured at small scales (≤1 km; [15]), whereas a strong association with *Pipistrellus* roost presence was detected at all scales studied (0.5–6 km). Many other variables retained this ability to predict the presence of a roost across the range of scales, indicating that bats can be sensitive to habitat modifications considerable distances from the actual roost site. Nevertheless, and as expected, the maps in this study share many similarities with those in Bellamy, Scott & Altringham [15], reflecting the fact that home foraging range of most bat species is small and close to their primary roost sites.

Where variables showed interspecific differences in their associations with roost presence, this could not be related simply to home range or body size. For instance, *M*. *daubentonii* and *N*. *noctula* roost presence were best predicted by habitat variables measured at small spatial scales (500–1,000 m), yet the large *N*. *noctula* will frequently fly a few kilometres from their roosts to forage [36], whereas the smaller *M*. *daubentonii* only occasionally forages over these distances [37].

The models revealed species- and scale-specific habitat associations with important implications for conservation. The habitat variables associated with human development, manmade surface and building cover, had strikingly varied effects on species roosting habitat suitability (Fig 4). Our study encompassed only small towns and villages: Kendal, to the east of the Park, has a population of around 27,500. None of those within the park exceeds 5,500 and most have several hundred or fewer inhabitants. Despite this, *M*. *nattereri*, *P*. *auritus* and *M*. *brandtii/mystacinus* were predicted to avoid these areas for roosting. All had negative associations with manmade surface and building cover, and one of these variables contributed most to these species’ final roost models. Built up areas themselves were poor roosting habitat for *P*. *auritus* and *M*. *brandtii/mystacinus*, and *M*. *nattereri* was strongly affected by human-altered landscapes over greater scales. The probability of presence declined with increasing building cover at the 4,000 m scale, suggesting *M*. *nattereri* will avoid even the fringes of development. The slow, low flight of *P*. *auritus* and *M*. *nattereri* may make them more vulnerable to predation in well-lit areas and the noise and danger of vehicle collision along major roads create a barrier [38][39][40][41][42]. Other species did not show a strong relationship with manmade areas and *P*. *pipistrellus* even had a positive association with road cover at all spatial scales, with road cover at 500 m contributing to the final model. *P*. *pipistrellus* uses the mainly narrow, minor roads, with low evening traffic levels and hedgerows or trees on both sides, for foraging [15][43]. These findings support the theory that more generalist bat species are better able to exploit built up environments, which offer a wide variety of potential roost buildings (e.g. [38][44][45]).

Our findings suggest that gleaning, woodland species with slow flight are particularly sensitive to urban development. Multivariate analysis showed that the negative effects of high road and building cover on roost presence (at medium to large scales) were largely independent of the provision of more suitable habitats. Large scale urbanisation will therefore reduce and fragment suitable habitat for these species, potentially leading to population decline, loss of the more specialist species, a decrease of species diversity around urban areas, and ultimately shifts in species distributions [44][46]. The *Pipistrellus* models indicated that these generalist species were dependent on the composition of the landscape at large spatial scales, suggesting that the provision of water, tree lines and deciduous tree cover within towns will improve their use by these species. A (small) town’s suitability will be further enhanced by high cover of deciduous and ancient woodland at its fringes, including at least one large block of deciduous woodland.

Woodland was an important resource for roosting bats in general (Fig 4). At the 500 m scale, the provision of even a small amount of deciduous woodland (5–20% cover) dramatically increased the probability of roost presence, in agreement with Boughey *et al*. [13]. The probability of finding a *P*. *auritus* roost building rose with an increase in the surrounding woodland edge density, the cover of ancient and deciduous woodland, and size of the maximum patch of woodland. This was true at all spatial scales, indicating that large scale deciduous woodland availability benefits this species. This supports other studies that found *P*. *auritus* to be heavily dependent on deciduous woodland cover [13][47].

Boughey *et al*. [13] concluded that the provision of networks of deciduous woodland patches, separated by a maximum distance of 440 m (90% of the roosts they analysed were within this distance from woodland), would be beneficial for the six UK bats species modelled. This analysis also illustrated that bats roost close to woodlands, with the maximum distance recorded for *P*. *pipistrellus* (535 m) and all other species roosting within a maximum of 383 m. Boughey et al.’s [13] national study did not find any significant relationships with woodland patch size, whereas our regional models suggest that *P*. *auritus*, *M*. *nattereri* and *Pipistrellus* species presence will be highest in areas where there is at least one large patch of woodland. This was most important within 1,000–2,000 m of the roost for *M*. *nattereri*, and 500 m for *P*. *auritus*. However, the presence of large blocks of woodland continued to improve *P*. *auritus* and *Pipistrellus* species’ roosting habitat suitability up to 6,000 m from the roost. The importance of landscape connectivity and habitat structure to insectivorous bats has also been highlighted by studies in continental Europe [48][49].

Coniferous woodland had a mixed effect on species’ roosting habitat suitability. *Pipistrellus* appeared to avoid this habitat for roosting, which resulted in large pockets of unsuitable roosting habitat on both species’ maps, regardless of the availability of other features positively related to roost presence. Other UK studies have not reported a significant negative correlation between *Pipistrellus* roost presence and coniferous woodland cover [13][50][51]. However, the use of coniferous woodland may depend on factors we did not consider, such as its relative abundance, tree density and species composition, and may also relate to the availability and configuration of other, more suitable habitats at larger scales. It should be noted that in the UK coniferous woodland is largely comprised of commercial plantations of non-native tree species, planted over the last 100 years, some of which are now managed in part for recreation and biodiversity.

### Conclusions and applications

This study has demonstrated that multi-scale HSM can provide detailed information on a species’ distribution and the habitat features that determine it at different scales. The models have scientific and practical value. They improve our understanding of large-scale habitat use by bats and allow us to model the effects of climate change, infrastructural development and agricultural change. In practical conservation they can be used to identify the least damaging sites for new developments, make informed decisions on impact and mitigation and for large and small scale opportunity mapping by identifying the best locations for new habitat and green corridors. The models can also identify diversity hotspots and the location of rare or vulnerable species or reintroduction sites. This spatially explicit, landscape scale approach is vital if we are to make effective decisions that protect biodiversity in landscapes under pressure from development and a changing climate.

We have developed an ArcGIS toolkit (*MultiScaleMaxentToolbox*) to facilitate the production of multi-scale EGVs for modelling habitat suitability for other species and locations with MaxEnt, available for download at ArcGIS Online [http://www.arcgis.com/home/item.html?id=e406a43e6ba84512aaeaff3fb7c59ef2]. The approach can be applied to a wide range of taxa for which data are becoming increasingly available via record centres and online portals such as the National Biodiversity Network Gateway (https://data.nbn.org.uk) and the Global Biodiversity Information Forum (http://www.gbif.org/). There is also increasing availability of free, user-friendly, and peer-reviewed software with online support communities for preparing GIS data (e.g. QGIS, http://www.qgis.org/; R, www.r-project.org), and creating, testing, and interpreting habitat suitability models (e.g. MaxEnt and ENMTools). The technique is therefore readily and freely accessible to both academics and practitioners, and has the potential to become a standard tool for supporting landscape scale decision-making [15][52][53][54].

## Supporting Information

S1 File Methods. (DOCX)

S2 File Results. (DOCX)
