# Supplementary material for: Predicting Species Distributions Using Record Centre Data: Multi-Scale Modelling of Habitat Suitability for Bat Roosts
Source: PLoS One. 2015 Jun 8;10(6):e0128440. doi: 10.1371/journal.pone.0128440 (PMC4460044; doi:10.1371/journal.pone.0128440)
Supplement: S1 File — (DOCX) [file pone.0128440.s001.docx]

S1 Supporting Information

**Predicting species distributions using record centre data: multi-scale modelling of habitat suitability for bat roosts**

Chloe Bellamy & John Altringham

**Methods**

*GIS*

GIS data were compiled from multiple data sources (Table S1) and analysed using GIS techniques to create a series of environmental variables. GIS data for each site were verified when possible using recent aerial photographs. Each data set was modified using ArcGIS 10 to extract the information we required to build each habitat variable.

**Supporting Information Table S1**. **GIS data sources**

| **Data** | **Product name** | **Data type** | **Source** | **Date** | **Scale** |
| --- | --- | --- | --- | --- | --- |
| Digital Terrain Model | OS Land-Form PROFILE DTM | 10 m raster | EDINA Digimap, University of Edinburgh | Unknown | 1:10,000 |
| Base land cover map | OS MasterMap Topography Layer | Vector | EDINA Digimap, University of Edinburgh | 2006 | Urban 1:1,250  Rural 1:2,500  Moorland 1:10,000 |
| Ancient woodland | Ancient Woodland Inventory (Provisional) for England | Vector | Natural England | 1999 | Generally 1:50,000 |
| Mixed woodland | National Inventory of Woodland & Trees | Vector | Forestry Commission | 1993 - 1994 | 1:25,000 |
| Park boundary | Lake District National Park | Vector | Natural England | 2010 | Mixed scale |

*MultiScaleMaxEnt*

All variables were created using an ArcGIS toolbox created by the authors, which has been openly published online (<http://www.arcgis.com/home/item.html?id=e406a43e6ba84512aaeaff3fb7c59ef2>). All tools are written using ArcGIS ModelBuilder and users are able to open and review the underlying geoprocessing steps.

**Model settings**

We increased the regularisation multiplier to two for all species since models frequently overfit to training data (2,3). Either the mammal target group or the random selection of buildings was as the pseudoabsence dataset (Fig. S1.1). We did not allow MaxEnt to extrapolate predictions in these new environments, but forced the response curves to remain constant at both extremes of a variable’s range (clamping). We took an added measure to reduce the effects of any differences in the environments within the projection region, and subtracted the clamping values from the logistic projections (“fade by clamping”).

| 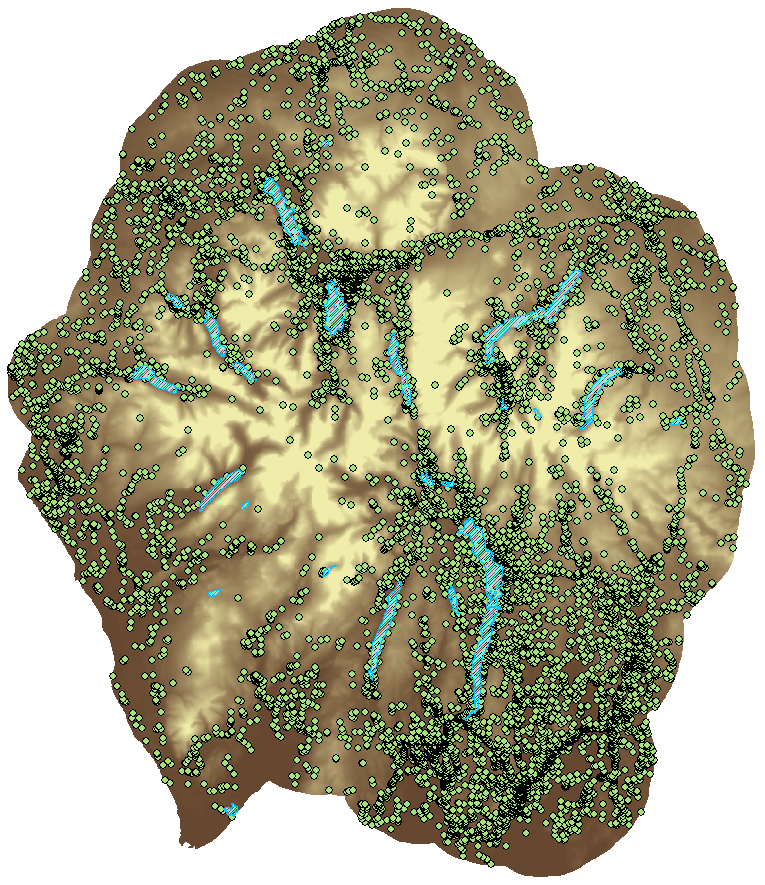  (a) | 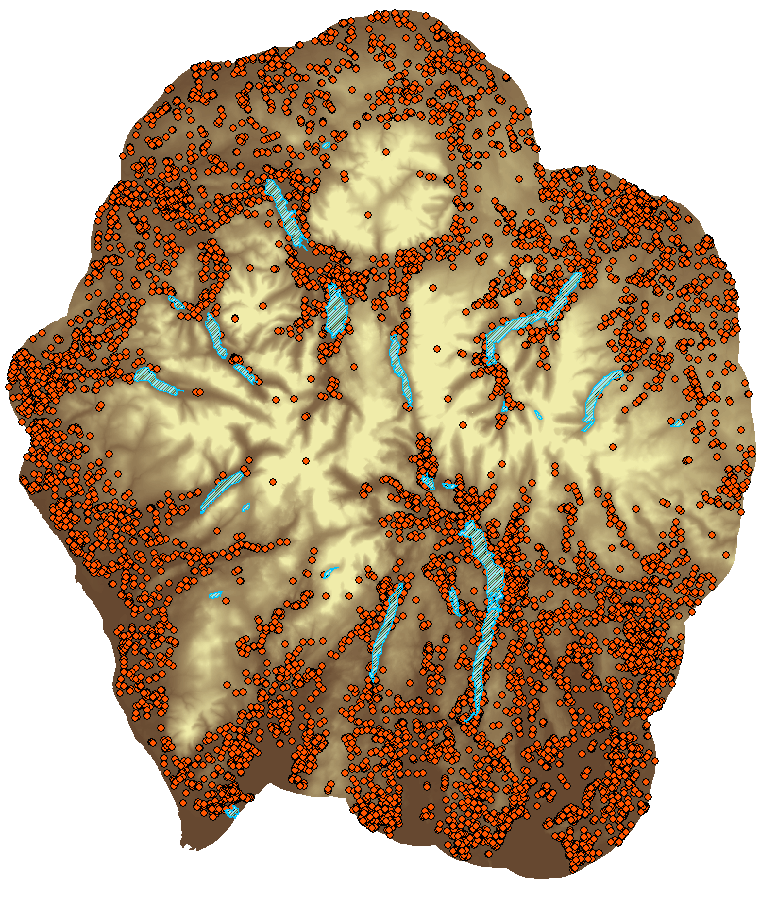  (b) |
| --- | --- |

Supporting Information Fig. S1.1. The distribution of the two sets of background data used to build roost habitat suitability models (a) Mammal records held by Tullie House Museum, (b) A random selection of 10,000 buildings from across the Park. Major lakes are shown in blue and the data is overlaid onto a digital terrain map displaying the range of altitudes across the Park (0 – 977 m, dark - light). © Crown Copyright/database right 2010. An Ordnance Survey/EDINA supplied service.

COMPARING SPECIES MAPS

A threshold-independent metric was applied to measure species’ niche breadth. These were estimated using ENMTools (www.ENMTools.com; 4, 5) by applying the inverse concentration metric of Levins (6). The degree of “niche overlap” (how closely two species share ecological niches) was also measured using ENMTools. Once each species’ HSI map had been standardised so that they summed to one over the entire study area, the *I* statistic was measured by calculating the difference in HSI at each raster cell between two species (4). HSI scores were also ranked across a species’ map to enable a non-parametric test of niche overlap, which compares cell-by-cell ranks between species pairs (Relative Rank (RR); 7). Both of these measures are standardised so that they range from zero to one – zero indicating no niche overlap and one indicating identical predicted distributions.

Species’ habitat suitability maps were combined in ArcGIS to generate richness maps. Each species’ binary ‘suitable/unsuitable’ habitat maps were overlaid to estimate the number of species for which a cell is predicted to be suitable using the ‘maximum sum of test sensitivity and specificity’ rule. These binary maps were overlaid rather than the continuous Habitat Suitability Indices because of the difficulties in interpreting and comparing the sum of continuous values.

**References**

| 1. | Smith S & Gilbert J (2003) The National Inventory of Woodland and Trees – Great Britain. Forestry Commission Inventory Report. Forestry Commission, Edinburgh. |
| --- | --- |
| 2. | Elith J, Kearney M & Phillips S (2010) The art of modelling range-shifting species. Methods in Ecology and Evolution 1: 330-342. |
| 3. | Anderson RP Gonzalez Jr (2011) Species-specific tuning increases robustness to sampling bias in models of species distributions: an implementation with Maxent. *Ecological Modelling* 222: 2796-2811. |
| 4. | Warren DL, Glor RE & Turelli M (2008) Environmental niche equivalency versus conservatism: quantitative approaches to niche evolution. Evolution 62: 2868-2883. |
| 5. | Warren DL, Glor RE & Turelli M (2010) ENMTools: a toolbox for comparative studies of environmental niche models. Ecography 33 (3): 607–611. |
| 6. | Levins R (1968) Evolution in changing environments. Monographs in Population Biology, Volume 2: Princeton University Press, Princeton. |
| 7. | Warren DL & Seifert SN (2011) Ecological niche modeling in Maxent: the importance of model complexity and the performance of model selection criteria. Ecological Applications 21: 335–342. |
