# Supplementary material for: Predicting Species Distributions Using Record Centre Data: Multi-Scale Modelling of Habitat Suitability for Bat Roosts
Source: PLoS One. 2015 Jun 8;10(6):e0128440. doi: 10.1371/journal.pone.0128440 (PMC4460044; doi:10.1371/journal.pone.0128440)
Supplement: S2 File — (DOCX) [file pone.0128440.s002.docx]

S2 Supporting Information

**Predicting species distributions using record centre data: multiscale modelling of habitat suitability for bat roosts**

**Results**

Chloe Bellamy & John Altringham

**Supporting Information Table S2.1. Bat species records for Cumbria 1980 – 2009 provided by Cumbria Biodiversity Data Centre (CBDC).** The first column shows all records of a known species/species group made at any time of the year in Cumbria. Reliable roost records are those confirmed as the likely site of a known species’ summer bat roost in Cumbria. Added records are the locations of known species roosts we identified which were not already in the CBDC database. The proportion of records belonging to each species is shown in brackets.

| Species | All data | Reliable roost records | Added |
| --- | --- | --- | --- |
| *Pipistrellus pipistrellus* | 561 (25.2%) | 188 (25.8%) | 2 |
| *P. pygmaeus* | 365 (16.3%) | 133 (18.2%) | 1 |
| *Nyctalus noctula* | 185 (8.3%) | 20 (2.7%) | 0 |
| *Myotis brandtii / mystacinus* | 187 (8.4%) | 68 (9.3%) | 1 |
| *M. daubentonii* | 311 (14.0%) | 96 (13.2%) | 3 |
| *M. nattereri* | 161 (7.2%) | 46 (6.3%) | 2 |
| *Plecotus auritus* | 457 (20.5%) | 179 (24.5%) | 7 |
| TOTAL | 2,227 | 730 | 16 |

**Supporting Information Table S2.2.** **The roost types identified for habitat suitability modelling**. Roost data were extracted for a 5 km buffer around the Park and classified according to the structure a bat was found to roost in (building = any building; CBDC box = data on roosting bats identified in bat boxes, supplied by CBDC; FC box = bats identified roosting in bat boxes on Forestry Commission land; bridge = bridges or tunnels; Other = any other structure). Records highlighted in bold were used for modelling. The proportion of total roost records belonging to each species is shown in brackets. Target group identifies the type of pseudoabsence data used to create a species’ model.

| Species | Building | Tree | CBDC box | FC box | Bridge | Other | Total | Total used | Target group |
| --- | --- | --- | --- | --- | --- | --- | --- | --- | --- |
| *P. pipistrellus* | **129** | 0 | 1 | 0 | 0 | 0 | 130 (30.1%) | **129** | Buildings |
| *P. pygmaeus* | **79** | 1 | 2 | 0 | 1 | 0 | 84 (19.4%) | **80** | Buildings |
| *N. noctula* | 0 | **6** | **1** | **2** | 0 | **1** | 10 (2.3%) | **10** | Mammal |
| *M. brandtii / mystacinus* | **24** | 0 | 0 | 0 | 2 | 0 | 26 (6.0%) | **24** | Buildings |
| *M. daubentonii* | **6** | **2** | 0 | 0 | **43** | 0 | 51 (11.8%) | **51** | Mammal |
| *M. nattereri* | **16** | 0 | 0 | **2** | **5** | 0 | 23 (5.3%) | **23** | Mammal |
| *P. auritus* | **102** | 0 | 2 | 1 | 3 | 0 | 108 (25.0%) | **102** | Buildings |
| TOTAL | 358 | 9 | 6 | 5 | 54 | 1 | 433 | **418** |  |

**Multivariate model composition**

**Supporting Information Table S2.3. The mean distance a species roost was to water or woodland edge.** Maximum distances recorded are shown in brackets. The average values for the pseudoabsences are shown for comparison (bold text).

| Species | Mean distance (m) to woodland edge ± S.D. (max) | Mean distance (m) to water ± S.D. (max) |
| --- | --- | --- |
| *P. pipistrellus* | 64.9 ± 72.7 (535.1) | 233.8 ± 194.6 (835.2) |
| *P. pygmaeus* | 49.6 ± 67.3 (382.9) | 152.6 ± 134.7 (511.9) |
| *P. auritus* | 48.3 ± 59.2 (347.3) | 216.2 ± 171.4 (786.9) |
| *M. brandtii / mystacinus* | 47.5 ± 46.4 (155.7) | 213.0 ± 140.5 (510.8) |
| Random building | **103.8 ± 131.9 (2,707.9)** | **260.9 ± 244.8 (1,868.8)** |
| *M. nattereri* | 48.6 ± 52.8 (155.7) | 134.1 ± 144.1 (449.1) |
| *N. noctula* | 19.6 ± 13.8 (50.7) | 209.5 ± 155.5 (499.13) |
| *M. daubentonii* | 42.4 ± 46.4 (206.8) | 42.3 ± 81.9 (535.1) |
| Mammal background | **95.7 ± 373.5 (2,754.5)** | **224.6 ± 315.7 (1,960.5)** |

Species tended to be associated with low altitudes at all scales, but responses to slope varied. At small spatial scales, the probability of finding a *P. auritus*, *M. brandtii*/*mystacinus*, or *P. pygmaeus* roost increased with slope, whereas the probability of other species’ presence peaked at 2 - 10°. At larger scales, all species were associated with shallow average slopes. Aspect was a useful predictor for *M. nattereri*, M*. brandtii*/*mystacinus* and *P. pygmaeus*. The probability of finding roosts of these species was highest on easterly aspects.

Roost presence was positively associated with deciduous, ancient and mixed woodland cover (Figure 4). Deciduous woodland cover was a useful, positive predictor of roost presence at small spatial scales for all species. At the 500 m scale, even a small amount of deciduous woodland (~5% cover) dramatically increased the probability of roost presence. Deciduous woodland cover retained its predictive power at large spatial scales for *Pipistrellus* species and *P. auritus*. There was a mixed response to coniferous woodland. At the strongest scales of association the probability of presence declined for *P. pipistrellus* (6,000 m) and *M. daubentonii* (2,000 m) with increasing coniferous woodland cover. *P. pygmaeus* (1,000 m) probability of presence peaked at 15% before declining to zero at 70% cover. The chance of finding a *P. auritus* roost increased with coniferous woodland cover at the 6,000 m scale, but peaked at approximately 50% at the 1,000 m scale. Relationships with water cover were also mixed. *M. daubentonii*, *M. nattereri* and *P. pygmaeus* were most strongly and positively associated with this habitat at small scales (500 – 1,000 m), whereas *P. auritus* and *M. brandtii*/*mystacinus* became more strongly related to water cover at larger scales.

Woodland edge density was useful as a positive predictor of roost presence for *N. noctula* and *M. daubentonii* only at small spatial scales, but it remained a useful, positive predictor at all scales for *Pipistrellus* and *P. auritus*. The presence of large patches of woodland increased the likelihood *of M. nattereri*, *Pipistrellus* and *P. auritus* presence. The availability of even small patches of woodland dramatically increased roosting habitat suitability at all scales (Figure 4).

*P. pipistrellus* was the only species whose presence was positively related to manmade surface cover, a relationship that maintained its strength across the scales. Increases over 20% manmade cover reduced all other species’ habitat suitability at scales ≤2,000 m, and this threshold decreased to 5 – 10% cover at larger scales, highlighting the negative impact of both small and large scale urbanisation on bats (Figure 4). *P. pipistrellus* was also positively associated with buildings at small spatial scales, whereas *Myotis* and *P. auritus* response curves generally peaked at low building cover at all scales (~10 – 20% cover). *M. brandtii*/*mystacinus* and *M. nattereri* appeared to be the most adversely affected by large scale urban cover (Figure 4).

*Pipistrellus* and *P. auritus* presence were positively related to areas rich in habitat types at most spatial scales (Figure 4). In contrast, *M. daubentonii* roosts were negatively associated with habitat richness at all scales.

**Multivariate model composition**

**Supporting Information Figure S2.1 Marginal response curves and variable contribution (%) to each species’ pruned model.** Graphs show the probability of a species’ presence (*p*) over a variable’s range, whilst other variables are held constant. The spatial scale each variable was measured at is indicated in brackets.

a) *P. pipistrellus* b) *N. noctula*

c) *P. pygmaeus*

d) *M. mystacinus / brandtii*

e) *M. daubentonii*

f) *M. nattereri*

g) *P. auritus*

COMPARING SPECIES MAPS

*P. pipistrellus* and *P. auritus* had the largest area of suitable roosting habitat across the study area (Table 3). In comparison, *N. noctula* and *M. daubentonii* were restricted in the area predicted to be suitable for roosting. *M. daubentonii* and *P. pygmaeus* had the narrowest niche breadths, *P. pipistrellus* and *P. auritus* the broadest (Table S2.4). Despite these differences, there was generally a high degree of niche overlap (Table S2.5). When measured using the *I* statistic, *P. auritus* had the greatest overlap with other species, *M. daubentonii* the least. Estimations of niche overlap using RR, on the other hand, suggested that the *N. noctula* habitat suitability map had the least overlap with other species’ maps and *M. nattereri* and *M. brandtii/mystacinus* had similar niches.

**Supporting Information Table S2.4. Proportion of suitable area and niche breadth for each species.** Areas calculated using maximum sum of test sensitivity and specificity occupancy threshold. Niche breadth is calculated using the inverse of Levins metric (Levins 1968).

| Species | Suitable area (%) | Niche breadth |
| --- | --- | --- |
| *P. pipistrellus* | 14.7 | 0.607 |
| *P. pygmaeus* | 13.4 | 0.411 |
| *N. noctula* | 3.9 | 0.522 |
| *M. mystacinus / brandtii* | 11.1 | 0.431 |
| *M. daubentonii* | 6.7 | 0.160 |
| *M. nattereri* | 13.8 | 0.530 |
| *P. auritus* | 22.2 | 0.582 |

**Supporting Information Table S2.5. Measurements of niche overlap between the species’ models.** Measurements are made in terms of the “*I* statistic” (shaded grey) and the non parametric “Relative Rank” (RR; Warren, Glor, & Turelli 2008).

| *I* statistic | *P. auritus* | *M. daubentonii* | *M. mystacinus / brandtii* | *M. nattereri* | *N. noctula* | *P. pipistrellus* | *P. pygmaeus* |
| --- | --- | --- | --- | --- | --- | --- | --- |
| Relative rank |  |  |  |  |  |  |  |
| *P. auritus* |  | 0.718 | 0.905 | 0.903 | 0.936 | 0.908 | 0.882 |
| *M. daubentonii* | 0.728 |  | 0.727 | 0.667 | 0.690 | 0.709 | 0.791 |
| *M. mystacinus / brandtii* | 0.788 | 0.787 |  | 0.918 | 0.855 | 0.834 | 0.847 |
| *M. nattereri* | 0.729 | 0.702 | 0.819 |  | 0.860 | 0.828 | 0.813 |
| *N. noctula* | 0.670 | 0.633 | 0.673 | 0.598 |  | 0.919 | 0.892 |
| *P. pipistrellus* | 0.698 | 0.726 | 0.714 | 0.624 | 0.603 |  | 0.895 |
| *P. pygmaeus* | 0.709 | 0.77 | 0.727 | 0.656 | 0.623 | 0.727 |  |

*Species richness*

Overlaying the species’ continuous habitat suitability maps and binary suitable/unsuitable maps generated new maps that indicated the potential value of habitats across the Park to roosting bats in general (Figure S2.2). These maps show that there was a mutual avoidance of the central high fells by roosting bats. The southern lowlands provided the largest area of high quality roosting habitat, although towns and large areas of coniferous woodland in this region were predicted to provide suitable habitat for fewer species than the surrounding deciduous woodland and farmland.

**
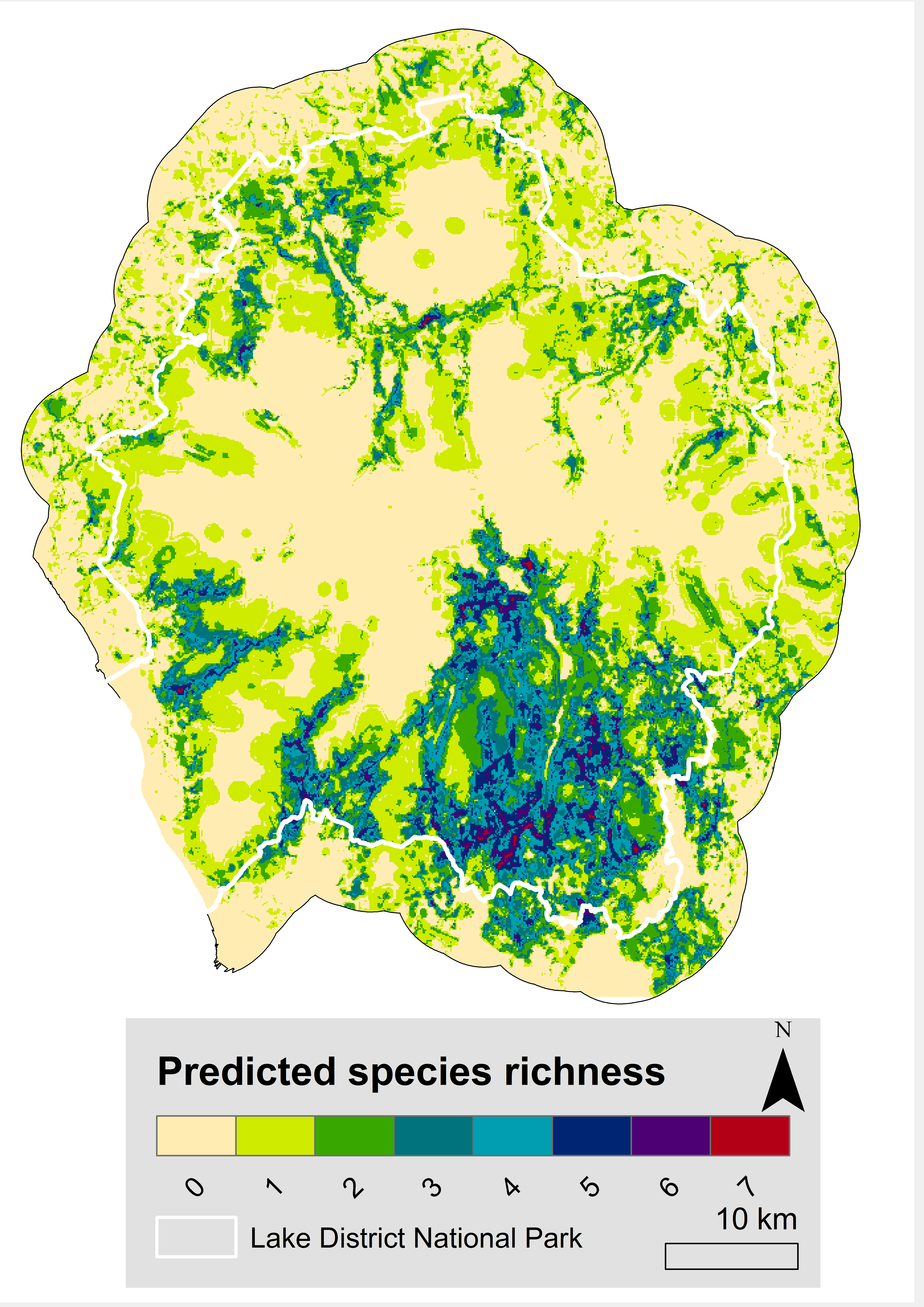
**

**Supporting Information Fig. S2. 2. Species richness map**. Number of species that a site provides suitable roosting habitat for, according to the maximum sum of sensitivity and specificity occupancy threshold rule. NB. *M. mystacinus* and *M. brandtii* are counted as one species. © Crown Copyright/database right 2010. An Ordnance Survey/EDINA supplied service.
